# Effects of Inherent Lactic Acid Bacteria on Inhibition of Angiotensin I-Converting Enzyme and Antioxidant Activities in Dry-Cured Meat Products

**DOI:** 10.3390/foods11142123

**Published:** 2022-07-18

**Authors:** Masaya Ogata, Jumpei Uchiyama, Abdulatef M. Ahhmed, Seiichi Sakuraoka, Satoshi Taharaguchi, Ryoichi Sakata, Wataru Mizunoya, Shiro Takeda

**Affiliations:** 1 Graduate School of Veterinary Science, Azabu University, Sagamihara 252-5201, Japan; da2101@azabu-u.ac.jp (M.O.); tsatoshi@azabu-u.ac.jp (S.T.); mizunoya@azabu-u.ac.jp (W.M.); 2 Department of Bacteriology, Graduate School of Medicine Dentistry and Pharmaceutical Sciences, Okayama University, Okayama 700-8558, Japan; uchiyama@okayama-u.ac.jp; 3 Department of Life Science Graduate School of Basic Sciences, The Libyan Academy of Graduate Studies, Janzour, Tripoli 79031, Libya; latef.ml@gmail.com; 4 Kyodo International, Inc., Kawasaki 216-0033, Japan; sakuraoka@kyodo-inc.co.jp; 5 School of Veterinary Medicine, Azabu University, Sagamihara 252-5201, Japan; sakata@azabu-u.ac.jp; 6 Center for Human and Animal Symbiosis Science, Azabu University, Sagamihara 252-5201, Japan

**Keywords:** dry-cured sausage, dry-cured ham, bioactivity, microflora, ACE inhibition, antioxidant activity, lactic acid bacteria

## Abstract

The aim of this study was to investigate the inherent bacteria that contribute to expressing the angiotensin I-converting enzyme (ACE) inhibitory activity and the antioxidant activity of dry-cured meat products without a bacterial starter. Among the ten dry-cured meat product samples, Coppa and Milano salami exhibited high ACE inhibitory activity, 2,2-diphenyl-1-picrylhydrazyl (DPPH) radical scavenging ability, and oxygen radical absorbance capacity (ORAC). No consistent trend was observed in the pH values or the total peptide and imidazole dipeptide concentration of the products that exhibited high ACE inhibitory and antioxidant activities in the tested samples. To investigate the bacteria contributing to the ACE inhibitory and antioxidant activities of the product, 16S rRNA sequencing analysis, isolation, and identification of bacteria were performed using not only Coppa and Milano salami but also the Jamon Serrano and Parma prosciutto products that had low functional activities. Results suggest the *Lactobacillales* order, particularly the species *Latilactobacillus sakei* and *Pediococcus pentosaceus*, were the main inherent bacteria in Coppa and Milano salami, respectively, compared with the Jamon Serrano and Parma prosciutto products. Therefore, the inherent lactic acid bacteria in dry-cured meat products without bacterial starter is important for ACE inhibitory and antioxidant activities of the products.

## 1. Introduction

Meat is generally considered to have high nutritional value; it is a good source of protein and contains B-vitamins, minerals, and trace elements. Given the increasing concern for human health, functional foods with health-promoting activity have garnered attention. The angiotensin I-converting enzyme (ACE) inhibitory activity and the antioxidant activities of meat and meat products have featured in many studies as potential bioactivities that can support human physiological functions. For example, ACE inhibition is proposed to result in antihypertensive activity, and antioxidant activity is proposed to control peroxidation reactions in the body; these functions can therefore contribute to the maintenance of good human health [1,2]. The bioactive peptides in meat can often demonstrate these activities. These bioactive peptides can be generated by cooking, depending on the acid/base conditions in which the protein is hydrolyzed (repulsion caused by an imbalance in charges), enzymatic hydrolysis, and microbial activity such as fermentation and ripening [3,4]. Thus, bioactivities such as ACE inhibition and antioxidant activity are an important quality of meat and meat products alongside their nutrition value.

Dry-cured meat products, including dry-cured ham, dry-cured loins, and fermented sausages, are well-known and highly appreciated products throughout Europe and many countries worldwide [5]. These products are made primarily from pork and then cured with salt and nitrite and/or nitrate. Some dry-fermented sausages have used a bacterial starter to assist the fermentation process. Then, a drying, ripening, and fermentation process is performed for several weeks, months, or even years. In some dry-cured meat products, peptides responsible for bioactivities such as ACE inhibition and antioxidant activity have been reported. There have been several reports on the effects of the LAB starter on bioactivity [6,7,8]. Moreover, several dry-cured meat products without using bacterial starter demonstrated bioactivities and their peptide and amino acid profiles were reported [9,10]. These profiles are thought to occur during meat processing. Since dry-cured meat products do not undergo any cooking process, the bacteria that participate in the processes from the environment or ingredients are naturally viable and present in the products. Thus, the inherent microorganisms in dry-cured meat products without a bacterial starter are thought to be particularly associated with the generation of bioactive peptides and bioactivities such as ACE inhibition and antioxidant activity [11]. Moreover, most of the inherent bacteria that contribute to the bioactivities of dry-cured meat products without using a starter are expected to vary because of the global diversity of cured meat products owing to the different manufacturing methods and ingredients used [12]. However, to the best of our knowledge, few reports have performed a detailed study of the inherent microorganisms in dry-cured meat products without a starter and investigated their effects on the expression of bioactivity in the products.

In recent years, metagenomic analysis, in which genomes are purified and sequenced directly from microbial communities, has been used to investigate microflora. Yang et al. demonstrated the relationship between microorganisms’ flora and flavor compounds and the nutrients in fermented meat products [13]. We hypothesized that differences in the bioactivity of dry-cured meat products could be due to the bacterial flora present in the products. Therefore, the aims of this study were to investigate the bacteria that contribute to expressing the bioactivity of dry-cured meat products, using a metagenomic analysis with 16s RNA amplicon sequencing. In this study, we investigated the biological characteristics, including ACE inhibition, 2,2-diphenyl-1-picrylhydrazyl (DPPH) radical scavenging (RS) ability, and oxygen radical absorbance capacity (ORAC) in 10 dry-cured meat products manufactured without a starter culture. Then, the bacterial flora in the products with and without these bioactivities were analyzed by 16s RNA amplicon sequencing. In addition, from the results of 16s RNA amplicon sequencing, several bacterial strains were isolated from those tested products, and the species were identified.

## 2. Materials and Methods

### 2.1. Materials

The dry-cured meat products used in this study are listed in Table 1. These products were manufactured without a bacterial starter culture. Each product was obtained through the Japan Cured Ham Association. The products were prepared from the three individual production lots. To maintain sample quality, they were stored at −20 °C before analysis, and then thawed to 4 °C overnight.

### 2.2. Assay of ACE Inhibitory Activity in the Products

Ten grams of each product was added to 40 mL of sterile distilled water, and the water extract was aseptically homogenized and sterile filtered out from the meat residue. The peptide concentration of the filtrates was measured by a reported method, with slight modifications [14]. The sample concentration was then adjusted to a peptide concentration of 10 mmol/L (Gly–Leu equivalent) (Gly–Leu, Tokyo Chemical Industry Co., Ltd., Tokyo, Japan) and the ACE inhibitory activity was tested. The ACE inhibitory activity assay utilized the fluorescence analysis method of Cheung et al. [15]. Briefly, 50 µL of sample solution, 100 µL of ACE 0.01 U/mL (Sigma Chemical Co., MO, USA), and 20 µL of 25 mM hippuryl-l-histidyl-l-leucine (Nacalai Tesque, Kyoto, Japan) were mixed in 96-well plate and incubated at 37 °C for 40 min. Subsequently, 100 µL of 1 M NaOH, 15 µL of 3.6 M phosphoric acid, and 10 µL of 0.2% *o*-phthaldialdehyde (OPA) (Nacalai Tesque) were added, and the sample fluorescence was measured under aseptic conditions using a POWERSCAN MX plate reader (DS Pharma Biomedical Co., Ltd., Osaka, Japan) with an excitation wavelength of 360 nm and emission wavelength of 460 nm. The inhibitory activity (%) was calculated using the following equation: Inhibitory activity (%) = ([Ac − As]/[Ac − Ab]) × 100, where Ac is the intensity of the control, As is the intensity of the sample, and Ab is the intensity of the blank. The data are presented as the inhibitory concentration 50 (IC_50_) for each sample.

### 2.3. Assay of 2,2-Diphenyl-1-Picrylhydrazyl (DPPH) Radical Scavenging (RS) Activity in the Products

First, 10 g of each product was added to 20 mL of sterile distilled water; then, the water extract was aseptically homogenized and sanitarily filtered out from the meat residue. The peptide content of the filtrates was determined [14] and the sample was adjusted to a peptide concentration of 20 mM (Gly–Leu equivalent) in 50% ethanol. The adjusted samples were subjected to a DPPH-RS activity assay in accordance with the method of Takeda et al. [7]. The DPPH-RS activity of the samples was evaluated as the reduction in absorbance of the DPPH radical (as detected at 520 nm) in the samples relative to the absorbance in the blank control sample. Trolox was used as the positive control and standard because it is a stable antioxidant that is widely used as an index of antioxidant activity. The data are expressed in moles of Trolox equivalent per gram for each sample.

### 2.4. Hydrophilic Oxygen Radical Absorbance Capacity (H-ORAC) of the Products

The H-ORAC of the products was determined using reported methods [16,17] with slight modification. Briefly, the samples were extracted in acetic acid–acidified aqueous methanol (methanol:water:acetic acid = 90:9.5:0.5 volumes) (MWA), and the extracts were diluted 10-fold with 75 mM potassium phosphate buffer (pH 7.4). The MWA-diluted samples, 6.0 μM fluorescein solution, and 31.7 mM 2,2′-azobis(2-amidinopropane) dihydrochloride (AAPH) were incubated in the assay buffer at 37 °C in a 96-well plate. The fluorescence (excitation at 485 nm, emission at 520 nm) was monitored every 2 min for 90 min using a POWERSCAN MX (DS Pharma Biomedical Co., Ltd.). The fluorescein intensity net of the area under the curve (AUC) was calculated by subtracting the AUC of the blank from the AUC of the sample or standard. The H-ORAC for each sample was determined by comparing its net AUC with that of the Trolox standard. Data are expressed as moles of Trolox equivalent per gram of sample.

### 2.5. Determination of pH, Free Peptides, and Imidazole Dipeptides in the Meat Products

The pH of products was directly measured by a pH meter (Testo K. K., Yokohama, Japan). For peptide analysis, 10 g of each product was added to 20 mL of sterile distilled water, and the water extract was aseptically homogenized and sanitarily filtered from the meat residue. Free peptides were measured by OPA reagent [14]. To determine the molecular weights of the components in the water extract, sodium dodecyl sulfate-polyacrylamide gel electrophoresis (SDS-PAGE) was performed on a 16% acrylamide gel and the gels were stained with Coomassie Brilliant Blue solution (Bio-Rad, CA, USA). Anserine and carnosine were measured as imidazole dipeptides by HPLC. Briefly, 0.5 mL of the supernatant of each water extracted sample was filtered by ultracentrifugation at 12,000 × *g* for 20 min at 4 °C to obtain the <10 kDa fractions (Nanosep 10K OMEGA; Pall Corp., NY, USA). Each of the obtained fractions was adjusted to 0.5 mL and analyzed with the HPLC Agilent SERIES 1100 system (Agilent Technologies Inc., CA, USA). For the analysis of anserine and carnosine, the test solution was injected into a reversed-phase column (InertSustain AQ-C18; GL Sciences Inc., Tokyo, Japan). Elution was performed at 30 °C with 0.2 M ammonium dihydrogen phosphate, 0.1 mM 1-pentanesulfonic acid sodium salt, and 4% acetonitrile solution adjusted to pH 2.0 with HCl; the flow rate was 0.8 mL/min. Anserine and carnosine were detected at an absorbance of 220 nm. A solution containing 5.0 mM L-anserine nitrate (Fujifilm Wako Pure Chemical) and 5.0 mM carnosine (*β*-alanyl-l-histidine, Peptide Institute, Inc., Osaka, Japan) was used as the standard.

### 2.6. Bacterial Communities in the Products

To extract the bacterial DNA from the tested meat products, three different production lots were prepared. Three slices from each lot of product were cut and ground individually in a mortar. The paste (0.5 g) of paste and sterile water (1.0 mL) were placed in a tube filled with glass beads (No. 2, Sansyo Co., Ltd., Tokyo, Japan) and shaken at 400 rpm for 30 s using a Micro Smash MS-100 (Tomy Seiko Co., Ltd., Tokyo, Japan). Then, centrifugation was performed at 1,000 rpm for 1 min. Finally, 200 μL of the supernatant was subjected to a bacterial DNA extraction protocol using bead beating (Nippon Gene, Tokyo, Japan). The concentration and purity of the extracted DNA solution were measured. The V3–V4 region of the 16S rRNA gene was amplified from the extracted DNA by two-step tailed PCR and the sequencing library was prepared using a kit (Illumina K.K., Tokyo, Japan). The purified amplicons were subjected to a paired-end sequencing using the MiSeq system and MiSeq Reagent Kit (Illumina K.K.). Library sequencing was performed by Anicom Specialty Medical Institute Inc. (Tokyo, Japan). The data were analyzed for microbial diversity using the Quantitative Insights Into Microbial Ecology (QIIME) 2 (version 2021.2). Sequence quality control and feature table construction were completed using the Divisive Amplicon Denoising Algorithm 2 (DADA2) QIIME 2 plugin. After the denoising step, a pretrained bacterial classifier was used to explore the taxonomic distribution of the samples. This classifier was trained using SILVA database version 138. Alpha diversity was calculated with the Shannon index. Beta diversity was estimated using the unweighted UniFrac distance.

### 2.7. Bacterial Counts and Identification of the Colonies Formed from the Products

To count the microbes in the tested products, the meat products were cut using a sterile knife and 1 g of sample and 9 mL of sterilized 0.85% saline were homogenized in a homogenizer (Nihonseiki Kaisha Ltd., Tokyo, Japan) at 12,000 rpm for 5 min. Serial dilutions (10^−1^–10^−8^) were prepared and 100 μL was spread on the agar plates. For the aerobic plate count, the standard agar method (Nissui Pharmaceutical Co., Ltd., Tokyo, Japan) was used and the samples were incubated at 37 °C for 2 days. For the LAB count, plate count agar with bromocresol purple (BCP) (Nissui Pharmaceutical Co.) was used and incubated at 37 °C for 3 days. In addition, glucose yeast peptone (GYP) agar [18] was used to count and isolate the LAB, which were incubated anaerobically at 37 °C for 3 days with anaerobic packs and jars (Mitsubishi Gas Chemical, Tokyo, Japan). The colonies on the GYP agar were purified by streak plating onto new plates of the same agar. Purified bacterial strains were suspended in 10% glycerol solution and stored at −80 °C. The strains were cultured with GYP broth and identified based on the 800 base pair (bp) sequences of the 5′-end of the 16S ribosomal RNA gene. DNA was extracted from bacterial colonies using bead beating (Nippon Gene). For DNA extraction, bead beating was performed at 5000 rpm using a Micro Smash MS-100 (Tomy Seiko Co., Ltd.). PCR was performed using the Prime Taq DNA Polymerase kit (GENETBIO, Daejeon, Korea) in a thermal cycler (TP350, TAKARA BIO INC., Kusatsu, Japan) in accordance with the manufacturers’ instructions. The following primers were used: forward, 5′-GTTTGATCCTGGCTCA-3′; reverse: 5′-TACCAGGGTATCTAATCC-3′. The cycling program consisted of initial denaturation (50 °C for 2 min and 95 °C for 10 min), followed by 40 cycles of 95 °C for 1 min, 55 °C for 1 min, and 72 °C for 2 min, with a final extension at 72 °C for 5 min. Amplified and purified 16S rDNA was sequenced by DNA sequence analysis at Fasmac Co., Ltd. (Atsugi, Japan). A homology search was performed using BLAST at the National Center for Biotechnology Information (https://blast.ncbi.nlm.nih.gov/Blast.cgi accessed on 10 June 2022). The LAB species were identified using BLAST results and a score higher than 98%.

### 2.8. Statistical Analysis

The results of ACE inhibitory activity, DPPH-RS ability, ORAC, pH values, bacterial relative frequency, and bacterial counts were analyzed by one-way ANOVA, with the Tukey–Kramer test used for multiple comparisons. GraphPad Prism software was used for the statistical analysis. The statistical analysis of microbial diversity was determined using the Kruskal–Wallis test and permutational multivariate analysis of variance (PERMANOVA) in QIIME 2. In this study, differences with *p*-values less than 0.05 were considered statistically significant.

## 3. Results

### 3.1. Bioactivities of the Dry-Cured Meat Products

**Figure 1:**
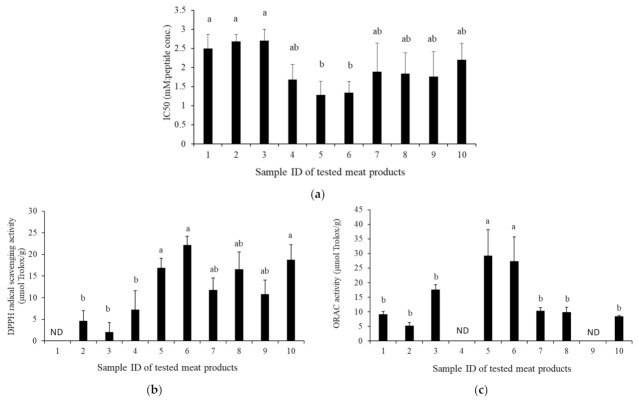
ACE inhibitory and antioxidant activities of the tested dry-cured meat products. (**a**) ACE inhibitory activity; (**b**) DPPH-RS ability, and (**c**) ORAC of the tested dry-cured meat products. Sample IDs: 1, 2, 3, 4, 5, 6, 7, 8, 9, and 10 represent Jamon Serrano, Black Forest ham, Parma prosciutto, Pancetta, Coppa, Milano salami, Parma salami, Salami with truffle, Longaniza, and Salchichón, respectively. Values with different lowercase letters indicate significant differences in each test (*p* < 0.05). ND, not detected.

The ACE inhibitory and antioxidant activities were reported to evaluate the bioactivity of dry-cured meat products [1,2]. The IC_50_ values of the ACE activity of the tested dry-cured meat products are shown in Figure 1a. The IC_50_ values of water-soluble extracts of Coppa (No. 5: shown in Figure 1) and Milano salami (No. 6) were almost the same, and lower than those of all the other products. In particular, they were significantly lower than those of the Jamon Serrano (No. 1), Black Forest ham (No. 2), and Parma prosciutto (No. 3) (*p* < 0.05). Thus, Coppa and Milano salami had the highest ACE inhibitory activities among the tested dry-cured meat products in this study.

The DPPH-RS ability and H-ORAC were investigated as the antioxidation of the tested samples; the results are shown in Figure 1b and Figure 1c, respectively. These activities are used as markers of the antioxidation activity of foods [19]. The DPPH-RS assay is a single electron transfer-based method, in which antioxidants reduce substrates by providing one electron to radicals and oxide; meanwhile, the ORAC assay is a hydrogen atom transfer-based method in which antioxidants inhibit substrate oxidation by providing hydrogen atoms to radicals and oxides [20]. The DPPH-RS ability of Milano salami was the highest among the tested products. The DPPH-RS abilities for Coppa, Milano salami, and Salchichón were significantly higher than those for Black Forest ham, Parma prosciutto, and Pancetta (*p* < 0.05). The DPPH-RS abilities for Coppa, Milano salami, and Salchichón were also higher than those for Parma salami, Salami with truffle, and Longaniza, but the difference was not significant. The H-ORAC of Coppa was the highest and the activity of Milano salami was the second highest among the tested samples; these values were significantly higher than the others (*p* < 0.05). Thus, Coppa and Milano salami had high antioxidant activity among the tested dry-cured meat products in this study.

### 3.2. pH and Peptide Content of the Dry-Cured Meat Products

**Figure 2:**
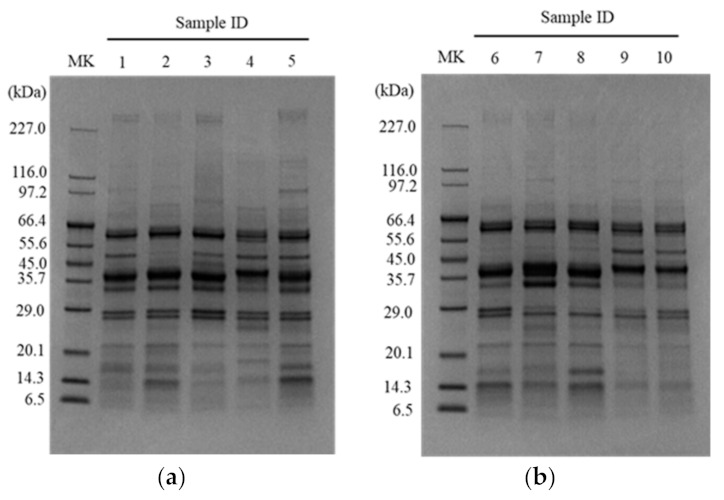
SDS-PAGE analysis of the water extracts of the tested dry-cured meat products. (**a**) SDS-PAGE analysis of Sample IDs 1, 2, 3, 4, and 5, which represent Jamon Serrano, Black Forest ham, Parma prosciutto, Pancetta, and Coppa, respectively. (**b**) SDS-PAGE analysis of Sample IDs 6, 7, 8, 9, and 10, which represent Milano salami, Parma salami, Salami with truffle, Longaniza, and Salchichón, respectively. MK, marker lane.

The pH values for each tested dry-cured meat product are shown in Table 2, which ranged from pH 5.74 (Milano salami) to pH 6.70 (Coppa). There was a significant difference between the pH of Milano salami and that of Coppa (*p* < 0.05). The water extracts of the individual lots of the tested products were pooled and analyzed for peptides and by SDS-PAGE. As shown in Table 2, the total peptide concentrations were higher in Parma prosciutto and Jamon Serrano, and those in Pancetta and Salchichón tended to be lower among the tested products. In addition, the imidazole peptides anserine and carnosine were measured. Anserine was below the limit of detection in the tested products, whereas carnosine was detected in all products, except for Salchichón slice (Table 2). The carnosine concentration in Parma prosciutto slice and Pancetta slice tended to be higher among the tested products. The results indicated that the total peptide and carnosine concentrations varied among the tested products. In the SDS-PAGE analysis of the dry-cured meat products, almost all bands were observed below 66.4 kDa (Figure 2). Lower and higher intensity bands at approximately 50 kDa and 14 kDa, respectively, were observed in Black Forest ham, Coppa, Milano salami, Parma salami, and Salami with truffle, but not in other products. Lower intensity bands were observed between 6.5 kDa and 29.0 kDa in each product. The expression patterns and intensities of these bands differed for each tested product, indicating that the rate of degradation of meat proteins by fermentation and/or ripening was different among the tested products.

### 3.3. Bacterial Communities in the Dry-Cured Meat Products

To investigate the major bacteria that contributed to the expression of bioactivity in the product, 16S rRNA sequencing analysis was carried out using Coppa and Milano salami, which showed the highest ACE inhibitory and antioxidant activities. In addition, sequencing analysis was performed on Jamon Serrano and Parma prosciutto on behalf of the products which showed low ACE inhibitory and antioxidant activities. After filtering in the sequencing samples, the library for each sample contained from 9087 to 30,223 sequences, and the amplicon sequence variants that were assigned as bacteria were yielded from 8467 to 30,025 sequences by applying DADA2 to the tested samples. Some samples were not subjected to taxonomic analysis or bacterial diversity analysis owing to an increase in nonchimeric reads after denoising.

**Figure 3:**
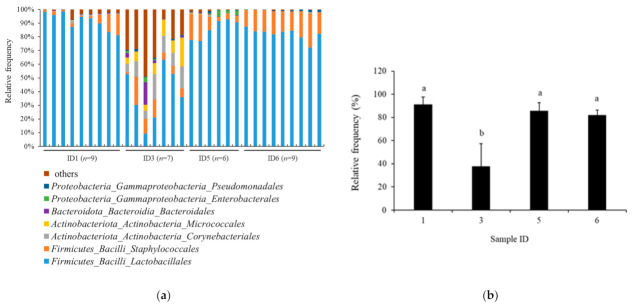
Bacterial community in the tested dry-cured meat products at the order level. (**a**) Bacterial taxa in the tested products. (**b**) Relative abundance of *Lactobacillales* order in the tested products. Values represent the mean ± SD. The different lowercase letters indicate significant differences (*p* < 0.05). Sample IDs 1, 3, 5, and 6 represent Jamon Serrano, Parma prosciutto, Coppa, and Milano salami.

The taxonomy analysis in the order of bacteria based on 16S rRNA gene sequencing is shown in Figure 3a for the Jamon Serrano (*n* = 9), Parma prosciutto (*n* = 7), Coppa (*n* = 6), and Milano salami (*n* = 9). *Lactobacillales* was clearly a major bacterial order, accounting for the largest proportion in the Jamon Serrano sample, followed by Coppa and Milano salami. However, the relative abundance of the Italian prosciutto was different from the Jamon Serrano, Coppa, and Milano salami, which were detected in the other bacterial orders, except for *Lactobacillales*. The Shannon index values for α-diversity for the samples were as follows: Jamon Serrano, 3.946 ± 0.249 (*n* = 9); Parma prosciutto, 5.914 ± 1.066 (*n* = 7); Coppa, 3.760 ± 0.353 (*n* = 6); and Milano salami, 3.057 ± 0.229 (*n* = 9). The *p*-value in the Kruskal–Wallis test for all groups was 9.46 × 10^−6^, which indicated a significant difference. The significant differences were also observed in all pairs by the Kruskal–Wallis test for pairwise, except for Jamon Serrano and Coppa pair (*p* < 0.05). Moreover, the comparison between the product groups with high and low bioactivities: the Jamon Serrano and Parma prosciutto group and the Coppa and Milano salami group, resulted in a *p*-value of 0.000205 by the Kruskal–Wallis pairwise test. Thus, the expression of bioactivities and the α-diversity of bacterial flora differed significantly between the meat products. In addition, significant differences were observed in the group significance plots in the PERMANOVA analysis of β-diversity (*p*-value was 0.001). The pairwise PERMANOVA results showed significant differences in all tested sample pairs (*p* < 0.05). Thus, it was suggested that the bacterial diversities of the tested dry-cured meat products, Jamon Serrano, Parma prosciutto, Coppa, and Milano salami, were remarkably different from each other. In addition, Figure 3b shows the relative abundance of *Lactobacillales* in the samples Jamon Serrano (*n* = 9), Parma prosciutto (*n* = 7), Coppa (*n* = 6), and Milano salami (*n* = 9). The *Lactobacillales* abundance of Parma prosciutto was significantly lower than those of Jamon Serrano, Coppa, and Milano salami (*p* < 0.05). 

**Figure 4:**
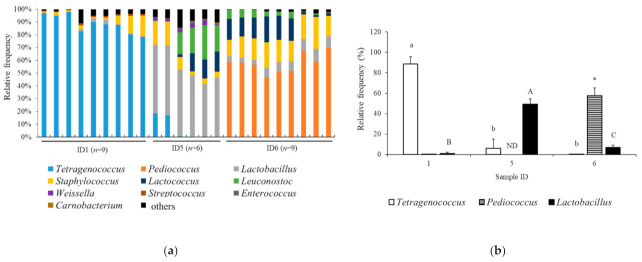
Bacterial community in the tested dry-cured meat products at the genus level. (**a**) Bacterial taxa in the tested products. (**b**) Relative abundance of the *Tetragenococcus*, *Pediococcus*, and *Lactobacillus* genera in the tested products. Values represent the mean ± SD. The different lowercase and capital letters indicate significant differences in *Tetragenococcus* and *Lactobacillus*, respectively (*p* < 0.05). The asterisks indicate significant difference in the level of *Pediococcus*, as compared to *Tetragenococcus* (*p* < 0.05). Sample IDs 1, 5, and 6 represent Jamon Serrano, Coppa, and Milano salami. ND, not detected.

The taxonomic analysis at the genus level in the samples Jamon Serrano (*n* = 9), Coppa (*n* = 6), and Milano salami (*n* = 9) is shown in Figure 4a, demonstrating the *Lactobacillales* levels determined by 16S rRNA gene sequencing. The major bacteria in the Jamon Serrano, Coppa, and Milano salami samples were in *Tetragenococcus*, *Lactobacillus*, and *Pediococcus* genera, respectively. The relative abundance of these genera in the Jamon Serrano, Coppa, and Milano salami samples is shown in Figure 4b. The abundance of *Tetragenococcus* in Jamon Serrano was significantly higher than in the other samples (*p* < 0.05). The abundance of *Lactobacillus* in Coppa was significantly higher than that in the other samples (*p* < 0.05). Moreover, the abundance of *Pediococcus* in Milano salami was significantly higher than in the other samples (*p* < 0.05). Thus, the ratio of bacteria belonging to *Lactobacillales*, especially *Lactobacillus* and *Pediococcus*, was the major bacterial flora in Coppa and Milano salami samples, which demonstrated high ACE inhibition and antioxidant activities.

### 3.4. LAB Count and Identification of the Forming Colony from the Dry-Cured Meat Products

The total viable bacterial count and viable LAB count of Jamon Serrano, Parma prosciutto, Coppa, and Milano salami were examined by culture method (Table 3). The total plate counts of the Coppa and Milano salami samples were significantly higher than those of the Jamon Serrano and Parma prosciutto samples (*p* < 0.05). In addition, the colony-forming unit count for the Jamon Serrano sample was the lowest, and this was significantly lower than the other samples (*p* < 0.05). For the LAB, the colony-forming unit count on the BCP agar plates for the Coppa and Milano salami samples was significantly higher than those for the Jamon Serrano and Parma prosciutto samples (*p* < 0.05). Moreover, colony formation was detected on the GYP agar plates of the Coppa and Milano salami samples, but not in the Jamon Serrano and Parma prosciutto samples. The number of viable LAB in the Coppa and Milano salami samples was higher than in the Jamon Serrano and Parma prosciutto samples. To determine the major LAB species isolated from the Coppa and Milano salami products, 10 colonies formed on the GYP agar plate were randomly selected and their species were analyzed. Eight of the ten strains in Coppa were identified as belonging to *Latilactobacillus* (*L*.) *sakei*; the other two strains belonged to *Enterococcus faecalis*. *L. sakei* was new-classified species from *Lactobacillus sakei* [21]. In contrast, in Milano salami, all 10 isolates belonged to *Pediococcus* (*P*.) *pentosaceus* (Table 4). The results of these isolated species were consistent with the trend in the genus-level analysis of 16S rRNA gene sequencing.

## 4. Discussion

Dry-cured meat products include dry-cured ham, dry-cured loin, and dry-fermented sausage [5]. To prepare the end products, the meats are not pasteurized by cooking but instead prepared by salting, smoking, and drying the meat. Consequently, they contain microbial communities of individual bacteria and fungi species [22]. Those microbes contribute to fermentation, proteolysis, lipolysis, and flavor formation of the individual products. Moreover, some products are reported to have various bioactivities, including ACE inhibition and antioxidant activity, which are expected to contribute to the promotion of human health and the improved quality of meat products.

We investigated the bioactivities in the ten dry-cured meat products without a bacterial starter (Table 1 and Figure 1). As shown in Figure 1, the bioactivities were measured, including ACE inhibitory activity, DPPH-RS ability, and ORAC. ACE plays an important role in the regulation of blood pressure in humans [1,2]. The antioxidant activities such as DPPH-RS ability and ORAC in meat products inhibit color deterioration, microbial growth, and lipid oxidation [23,24]. The ingestion of food with antioxidant activity is also thought to control peroxidative reactions in the body and contribute to the maintenance of good human health [25,26]. Coppa and Milano salami displayed the highest level of all tested biological activities in this study. The ACE inhibitory activity and antioxidant activity of dry-cured ham and dry-fermented sausages were reported previously [7,9,10], and our results were consistent with these reports. Thus, as shown by the results in this study, the extracts of Coppa and Milano salami have the potential to exert good antihypertensive and antioxidant activities.

As shown in Table 2, the pH value of the tested dry-cured meat products and the total peptides in the water-soluble extracts were measured. The pH of the Coppa was the highest (6.70 ± 0.12) and that of the Milano salami was the lowest (5.74 ± 0.09). According to the reference in a previous report, the pH of the meat products used in this study is consistent with most traditional meat products [27]. The ripening process leads to the hydrolysis of certain compounds, such as proteins and lipids, as well as the formation and release of low-molecular-weight compounds [5]. Moreover, because dry-cured meat products are not cooked, the natural microbes tend to live in the products. Thus, they affect the hydrolysis of the meat components and the generation of products, such as peptides. As shown in Table 2, the peptide concentrations in the water extracts of tested products were investigated, and Parma prosciutto was the highest (27.71 mM) and that of Pancetta was the lowest (4.96 mM). The total peptide concentrations in the Jamon Serrano and Parma prosciutto, whose ripening times were longer, tended to be higher among the tested product samples. The peptide concentrations of the Coppa (10.37 mM) and Milano salami (8.26 mM) samples, which demonstrated promising bioactivities, were in the middle range. In addition, the expression patterns and intensities of the SDS-PAGE bands were different for each of the tested products (Figure 2). In this study, the concentration of peptides extracted from the products tended to be dependent on the aging period. Then, the high bioactivities of the dry-cured meat products did not appear to correlate with the ripening period and the concentration of peptides released. Imidazole dipeptides, including anserine and carnosine, are known bioactive components of meat and display ACE inhibitory and antioxidant activities [28,29]. Carnosine was detectable in the tested products, but anserine was not (Table 2). The carnosine levels in the extracts of Coppa and Milano salami were low among the tested products, whereas the levels in Parma prosciutto and Pancetta slices were slightly higher. Overall, there were no consistent trends in the pH value, total peptide concentration, and imidazole dipeptide concentration of the products that demonstrated high ACE inhibitory activity and antioxidant activity in the current study. Many studies have shown that the ACE inhibitory activity and antioxidant activity of meat products are related to peptides derived from meat proteins that are generated during the fermentation and ripening processes [4]. Although we could not investigate the bioactive substances of the Coppa and Milano salami samples used in this study in detail, their potential activities might be affected by the differences in the constituent bioactive peptides that were generated by the fermentation and ripening processes.

The microbial communities were investigated in the samples of not only Coppa and Milano salami slices that showed bioactivities but also Jamon Serrano and Parma prosciutto that were low activities. According to the α diversity and β diversity determined by 16S rRNA gene sequencing, the bacterial diversity of the tested dry-cured meat products clearly differed from each other. The relationship between the expressed bioactivities and the α-diversity of bacterial flora significantly varied among the meat products. Coppa and Milano salami ripened faster than Jamon Serrano and Parma prosciutto (Table 1), which might influence the expression of bioactivities and their bacterial flora. Water activity is known to be an important intrinsic parameter that affects the viability of food microbes. However, as shown in Table 1, the water activities of the tested products, Jamon Serrano, Parma prosciutto, Coppa, and Milano salami, were almost the same, which seemed that water activity did not directly influence the bacterial flora composition of meat products in the present study. Dry-cured or cured meat products are globally diverse in terms of manufacturing methods and ingredients [12]. Thus, it was estimated that the bacterial flora in the tested products would also differ. The comparison of the major bacterial orders showed that the relative abundance of *Lactobacillales* in Parma prosciutto was significantly lower than that of Jamon Serrano, Coppa, and Milano salami (Figure 3). Parma prosciutto is the product with the longest ripening period in this study (Table 1), which is potentially caused to the low relative abundance of *Lactobacillales*. *Lactobacillales* is an order of bacteria in the Firmicutes phylum that mainly comprises LAB. Moreover, LABs are one of the typical types of bacteria in the European dry-cured meat products and they have been described to be the most active microbe in the acidification and denitrification processes, lipolysis, and proteolysis [10,30]. Hereby, the LABs in cured meat products would be important bacteria for acidification and denitrification processes, lipolysis, and proteolysis, as well as for the expression of bioactivities. Furthermore, the major bacterial genera in the Jamon Serrano, Coppa, and Milano salami samples were *Tetragenococcus*, *Lactobacillus*, and *Pediococcus*, respectively (Figure 4). The LAB counts in Coppa and Milano salami were significantly higher than in Jamon Serrano and Parma prosciutto, which was suggested to be a large number of LAB; notably, *Lactobacillus* and *Pediococcus* were the main inherent bacterial species, respectively (Table 3 and Table 4). The LAB count of dry-cured meat products that are inoculated with a LAB starter is approximately 8 log_10_ CFU/g [7,31]. The LAB count in the Coppa and Milano salami products used in this study was low because these products were processed without using a bacterial starter. The traditional production of fermented meat products is mostly based on spontaneous fermentation resulting from microbiota present naturally in the environment and raw materials, especially LAB [12,32]. For example, the production of traditional fermented sausages in Europe is based mainly on *Lactobacillus sakei*, *Lactobacillus curvatus*, and *Lactobacillus plantarum* [32,33,34]. In addition, *Pediococcus* was found in European dry-fermented meat products [35,36]. As shown in Table 4, the main LAB isolates from the Coppa and Milano salami samples were in *L. sakei* and *P. pentosaceus*, which confirmed the suggested results from 16S rRNA gene sequencing. Therefore, in this study, *L. sakei* and *P. pentosaceus* are suggested to be the main inherent LAB species in Coppa and Milano salami, and they would be important contributors toward the ACE inhibitory and antioxidant activities. The expressions of ACE inhibitory activities and/or antioxidation activity in meat products inoculated with LAB strains belonging to *L. sakei* and *P. pentosaceus* were reported in the previous studies [7,8,10]. In addition, *L. sakei* and *P. pentosaceus* have been used as a starter for processing fermented meat products [37]. Thus, as shown in this study, the isolates from Coppa and Milano salami in this study might be useful for processing the fermented meat products that demonstrated high ACE inhibitory activities and antioxidation activities. Further studies are required to investigate the application of LAB isolates in the current study for meat processing. Besides bacteria, yeasts and molds are involved in the fermentation and ripening of meat products, and their proteolytic enzyme activities are known to affect meat quality, such as its flavor and aroma [38]. Thus, further studies on the effects of inherent yeasts and molds on bioactivities are also warranted.

In conclusion, the ACE inhibitory activity, DPPH-RS ability, and ORAC, which are used to characterize the bioactivities of meat products, were investigated among the ten dry-cured meat products. The water-soluble extracts of Coppa and Milano salami were demonstrated to have low IC_50_ values for ACE inhibitory activity and high DPPH-RS ability and ORAC as antioxidant activity among the tested products. The total peptide and imidazole peptide concentrations of each sample were examined and found that the extracts of Coppa and Milano salami had neither particularly high nor low concentrations. In addition, the SDS-PAGE results showed that the expression patterns and intensities differed in each extract from the tested product. To investigate the correlation between the expression of bioactivity and the bacterial flora in the product, 16S rRNA gene sequencing was performed on the Coppa and Milano salami that exhibited high ACE inhibitory and antioxidant activities, and also on the Jamon Serrano and Parma prosciutto that had low ACE inhibitory and antioxidant activities. Then, the bacterial diversities of those products were clearly different from each other. In addition, the *Lactobacillales* order, in particular the *Lactobacillus* and *Pediococcus* genera, were the main inherent bacteria in the tested Coppa and Milano salami, respectively, and the LAB counts were higher than in the Jamon Serrano and Parma prosciutto. Moreover, LAB isolates from Coppa and Milano salami were frequently identified in *L. sakei* and *P. pentosaceus*. Therefore, the bioactivity levels in dry-cured meat products are different, and that variation might occur as a result of the unique bioactive peptides generated. In addition, the presence of bacteria belonging to the *Lactobacillales* order would be important for dry-cured meat products to express bioactivities, such as ACE inhibitory activity and antioxidant activity. In this study, the isolates belonging to *L. sake* and *P. pentosaceus* were thought to contribute to the ACE inhibitory and antioxidant activities. The production of cured meat products is diverse worldwide in terms of manufacturing methods and ingredients. For a more detailed identification of the inherent bacterial species associated with the expression of bioactivity in meat products, further studies are required to investigate the relationship between the levels of bioactivity and the major bacteria in more cured meat products. 

## Figures and Tables

**Table 1:** Tested dry-cured meat products.

Meat Products	Type	Processes of Salting and Post Salting	Ripening Time	Water Activity	Origin
Jamon Serrano slice	Dry cured ham	Covered salting and drying	9 months	0.91	Spain
Black Forest ham slice	Dry cured ham	Soaked salting, drying, and smoking	3 months	0.91	German
Parma prosciutto slice	Dry cured ham	Covered salting and drying	24 months	0.91	Italy
Pancetta slice	Dry cured belly	Covered salting and drying	1 week	0.92	Italy
Coppa slice	Dry cured ham	Covered salting and drying	1 month	0.92	Italy
Milano salami slice	Dry cured sausage	Mixed salting and drying	2 weeks	0.91	Italy
Parma salami slice	Dry cured sausage	Mixed salting and drying	1 week	0.90	Italy
Salami with truffle slice	Dry cured sausage	Mixed salting and drying	2 weeks	0.92	Italy
Longaniza	Dry cured sausage	Mixed salting and drying	2 weeks	0.91	Spain
Salchichón slice	Dry cured sausage	Mixed salting and drying	2 weeks	0.89	Spain

All products are manufactured without a bacterial starter culture.

**Table 2:** pH and peptide levels in the tested meat products.

	Dry-Cured Meat Products									
	Jamon Serrano	Black Forest ham	Parma Prosciutto	Pancetta	Coppa	Milano Salami	Parma Salami	Salami With Truffle	Longaniza	Salchichón
pH	6.05 ± 0.13 ^de^	5.92 ± 0.10 ^def^	6.07 ± 0.06 ^cd^	6.32 ± 0.15 ^b^	6.70 ± 0.12 ^a^	5.74 ± 0.09 ^f^	6.68 ± 0.07 ^a^	5.87 ± 0.03 ^df^	6.04 ± 0.04 ^de^	6.16 ± 0.13 ^bc^
Total peptide * (mM)	21.25	8.12	27.71	4.96	10.37	8.26	6.86	6.75	6.05	5.21
Carnosine (mM)	0.12	0.19	0.20	0.21	0.18	0.15	0.17	0.17	0.14	ND
Anserine (mM)	ND	ND	ND	ND	ND	ND	ND	ND	ND	ND

* The values are shown as Gly-Leu relative conc. The pH represents the mean ± SD of three independent experiments. Values with different superscript lowercase letters indicate significant differences between each other (*p* < 0.05). Total peptide, carnosine, and anserine data are the triplicate averages of the measurements from the water extracts pooled from three individual samples. ND means not detected by HPLC analysis.

**Table 3:** Bacterial counts in the tested dry-cured meat products.

Colony-Forming Count	Dry-Cured Meat Products			
(Log_10_ CFU/g)	Jamon Serrano	Parma Prosciutto	Coppa	Milano Salami
Total bacteria	2.21 ± 0.29 ^a^	3.75 ± 0.04 ^b^	4.61 ± 0.17 ^c^	4.48 ± 0.36 ^c^
Lactic acid bacteria				
BCP agar plate	2.27 ± 0.14 ^a^	3.95 ± 0.04 ^b^	4.21 ± 0.31 ^c^	4.66 ± 0.08 ^c^
GYP agar plate	ND	ND	3.67 ± 0.70 ^a^	4.63 ± 0.30 ^b^

The bacterial numbers represent the mean ±SD of three independent experiments. Values with different superscript lowercase letters indicate significant differences between each other in the rows (*p* < 0.05). BCP, bromocresol purple; GYP, glucose yeast peptone; ND, not determined.

**Table 4:** Lactic acid bacteria species isolated from the Coppa and Milano salami samples.

Isolated Strain Number	Coppa	Milano Salami
1	*Latilactobacillus sakei*	*Pediococcus pentosaceus*
2	*Latilactobacillus sakei*	*Pediococcus pentosaceus*
3	*Enterococcus faecalis*	*Pediococcus pentosaceus*
4	*Enterococcus faecalis*	*Pediococcus pentosaceus*
5	*Latilactobacillus sakei*	*Pediococcus pentosaceus*
6	*Latilactobacillus sakei*	*Pediococcus pentosaceus*
7	*Latilactobacillus sakei*	*Pediococcus pentosaceus*
8	*Latilactobacillus sakei*	*Pediococcus pentosaceus*
9	*Latilactobacillus sakei*	*Pediococcus pentosaceus*
10	*Latilactobacillus sakei*	*Pediococcus pentosaceus*

Ten colonies formed on the glucose yeast peptone agar were randomly selected and identified their species by 16S rDNA sequences.

## Data Availability

All data are contained within the manuscript.

## References

[1] Ahhmed A.M., Muguruma M. (2010). A review of meat protein hydrolysates and hypertension. Meat Sci..

[2] Toldrá F., Gallego M., Reig M., Aristoy M.C., Mora L. (2020). Recent progress in enzymatic release of peptides in foods of animal origin and assessment of bioactivity. J. Agric. Food Chem..

[3] Toldrá F., Reig M., Aristoy M.C., Mora L. (2018). Generation of bioactive peptides during food processing. Food Chem..

[4] López-García G., Dublan-García O., Arizmendi-Cotero D., Gómez-Oliván L.M. (2022). Antioxidant and antimicrobial peptides derived from food proteins. Molecules.

[5] Geiker N.R.W., Bertram H.C., Mejborn H., Dragsted L.O., Kristensen L., Carrascal J.R., Bügel S., Astrup A. (2021). Meat and human health-current knowledge and research gaps. Foods.

[6] Mora L., Escudero E., Aristoy M.C., Toldrá F. (2015). A peptidomic approach to study the contribution of added casein proteins to the peptide profile in Spanish dry-fermented sausages. Int. J. Food Microbiol..

[7] Takeda S., Matsufuji H., Nakade K., Takenoyama S., Ahhmed A., Sakata R., Kawahara S., Muguruma M. (2017). Investigation of lactic acid bacterial strains for meat fermentation and the product’s antioxidant and angiotensin-I-converting-enzyme inhibitory activities. Anim. Sci. J..

[8] Zhang Y., Hu P., Xie Y., Yang P., Zheng S., Tian Y., Li J., Feng D. (2020). DNA damage protection and antioxidant activities of peptides isolated from sour meat co-fermented by *P. pentosaceus* SWU73571 and *L. curvatus* LAB26. CyTA-J. Food.

[9] Mora L., Escudero E., Toldrá F. (2016). Characterization of the peptide profile in Spanish Teruel, Italian Parma and Belgian dry-cured hams and its potential bioactivity. Food Res. Int..

[10] Gallego M., Mora L., Escudero E., Toldrá F. (2018). Bioactive peptides and free amino acids profiles in different types of European dry-fermented sausages. Int. J. Food Microbiol..

[11] Toldrá F., Gallego M., Reig M., Aristoy M.C., Mora L. (2020). Bioactive peptides generated in the processing of dry-cured ham. Food Chem..

[12] Halagarda M., Wójciak K.M. (2022). Health and safety aspects of traditional European meat products. A review. Meat Sci..

[13] Yang P., Zhong G., Yang J., Zhao L., Sun D., Tian Y., Li R., Rong L. (2022). Metagenomic and metabolomic profiling reveals the correlation between the microbiota and flavor compounds and nutrients in fermented sausages. Food Chem..

[14] Church F.C., Swaisgood H.E., Porter D.H., Catignani G.L. (1983). Spectrophotometric assay using *o*-phthaldialdehyde for determination of proteolysis in milk and isolated milk proteins. J. Dairy Sci..

[15] Cheung H.S., Wang F.L., Ondetti M.A., Sabo E.F., Cushman D.W. (1980). Binding of peptide substrates and inhibitors of angiotensin-converting enzyme. Importance of the COOH-terminal dipeptide sequence. J. Biol. Chem..

[16] Watanabe J., Oki T., Takebayashi J., Yamasaki K., Takano-Ishikawa Y., Hino A., Yasui A. (2012). Method validation by interlaboratory studies of improved hydrophilic oxygen radical absorbance capacity methods for the determination of antioxidant capacities of antioxidant solutions and food extracts. Anal. Sci..

[17] Watanabe J., Oki T., Takebayashi J., Takano-Ishikawa Y. (2014). Extraction efficiency of hydrophilic and lipophilic antioxidants from lyophilized foods using pressurized liquid extraction and manual extraction. J. Food Sci..

[18] Takeda S., Yamasaki K., Takeshita M., Kikuchi Y., Tsend-Ayush C., Dashnyam B., Ahhmed A.M., Kawahara S., Muguruma M. (2011). The investigation of probiotic potential of lactic acid bacteria isolated from traditional Mongolian dairy products. Anim. Sci. J..

[19] Gulcin İ. (2020). Antioxidants and antioxidant methods: An updated overview. Arch. Toxicol..

[20] Huang D., Ou B., Prior R.L. (2005). The chemistry behind antioxidant capacity assays. J. Agric. Food Chem..

[21] Zheng J., Wittouck S., Salvetti E., Franz C.M.A.P., Harris H.M.B., Mattarelli P., O’Toole P.W., Pot B., Vandamme P., Walter J. (2020). A taxonomic note on the genus *Lactobacillus*: Description of 23 novel genera, emended description of the genus *Lactobacillus* Beijerinck 1901, and union of *Lactobacillaceae* and *Leuconostocaceae*. Int. J. Syst. Evol. Microbiol..

[22] Franciosa I., Alessandria V., Dolci P., Rantsiou K., Cocolin L. (2018). Sausage fermentation and starter cultures in the era of molecular biology methods. Int. J. Food Microbiol..

[23] Lauzurica S., De la Fuente J., Díaz M.T., Alvarez I., Pérez C., Cañeque V. (2005). Effect of dietary supplementation of vitamin E on characteristics of lamb meat packed under modified atmosphere. Meat Sci..

[24] Kurćubić V.S., Mašković P.Z., Vujić J.M., Vranić D.V., Vesković-Moračanin S.M., Okanović Đ.G., Lilić S.V. (2014). Antioxidant and antimicrobial activity of Kitaibelia vitifolia extract as alternative to the added nitrite in fermented dry sausage. Meat Sci..

[25] Dávalos A., Miguel M., Bartolomé B., López-Fandiño R. (2004). Antioxidant activity of peptides derived from egg white proteins by enzymatic hydrolysis. J. Food Prot..

[26] Dimitrios B. (2006). Sources of natural phenolic antioxidants. Trends Foods Sci. Technol..

[27] Ambrosiadis J., Soultos N., Abrahim A., Bloukas J.G. (2004). Physicochemical, microbiological and sensory attributes for the characterization of Greek traditional sausages. Meat Sci..

[28] Han Y., Gao B., Zhao S., Wang M., Jian L., Han L., Liu X. (2019). Simultaneous detection of carnosine and anserine by UHPLC-MS/MS and its application on biomarker analysis for differentiation of meat and bone meal. Molecules.

[29] Gil-Agustí M., Esteve-Romero J., Carda-Broch S. (2008). Anserine and carnosine determination in meat samples by pure micellar liquid chromatography. J. Chromatogr. A.

[30] Flores M., Toldrá F. (2011). Microbial enzymatic activities for improved fermented meats. Trend. Food Sci. Technol..

[31] Cardinali F., Milanović V., Osimani A., Aquilanti L., Taccari M., Garofalo C., Polverigiani S., Clementi F., Franciosi E., Tuohy K. (2018). Microbial dynamics of model Fabriano-like fermented sausages as affected by starter cultures, nitrates and nitrites. Int. J. Food Microbiol..

[32] Talon R., Leroy S., Lebert I. (2007). Microbial ecosystems of traditional fermented meat products: The importance of indigenous starters. Meat Sci..

[33] García Fontán M.C., Lorenzo J.M., Parada A., Franco I., Carballo J. (2007). Microbiological characteristics of “androlla,” a Spanish traditional pork sausage. Meat Sci..

[34] Pradoa N., Sampayoa M., Gonzáleza P., Lombób F., Díaza J. (2019). Physicochemical, sensory and microbiological characterization of Asturian Chorizo, a traditional fermented sausage manufactured in Northern Spain. Meat Sci..

[35] Benito M.J., Martín A., Aranda E., Pérez-Nevado F., Ruiz-Moyano S., Córdoba M.G. (2007). Characterization and selection of autochthonous lactic acid bacteria isolated from traditional Iberian dry-fermented Salchichón and chorizo sausages. J. Food Sci..

[36] Benito M.J., Serradilla M.J., Ruiz-Moyano S., Martín A., Pérez-Nevado F., Córdoba M.G. (2008). Rapid differentiation of lactic acid bacteria from autochthonous fermentation of Iberian dry-fermented sausages. Meat Sci..

[37] Ammor M.S., Mayo B. (2007). Selection criteria for lactic acid bacteria to be used as functional starter cultures in dry sausage production: An update. Meat Sci..

[38] Palavecino Prpich N.Z., Camprubí G.E., Cayré M.E., Castro M.P. (2021). Indigenous microbiota to leverage traditional dry sausage production. Int. J. Food Sci..

